# A single-center experience: management of patients with thymic epithelial tumors

**DOI:** 10.1186/s12957-020-01988-4

**Published:** 2020-08-13

**Authors:** Marius Kemper, Mona Moradzadeh, Eugen Bellon, Ahmad S. Bahar, Rainer Grotelüschen, Matthias Reeh, Jakob R. Izbicki, Kai Bachmann

**Affiliations:** Department of General, Visceral and Thoracic Surgery, University Medical Centre Hamburg-Eppendorf, Martinistrasse 52, 20246 Hamburg, Germany

**Keywords:** Thymic epithelial tumors, Masaoka, Thymectomy, Clavien-Dindo, Survival, Neoadjuvant therapy

## Abstract

**Background:**

Thymic epithelial tumors are rare neoplasias. There are no internationally accepted standards to treat this complex oncological disease. The studies on which our knowledge is based frequently have methodological weaknesses. If the tumor is resectable, complete surgical excision is currently the first-line therapy. Thymic epithelial tumors respond to radiation. The therapeutic benefit of adjuvant radiotherapy depends on tumor stage. To validate and improve treatment, we share our current experiences with clinical management and surgical intervention.

**Methods:**

This single-center retrospective study included 40 patients with primarily resectable thymic epithelial tumors who underwent resection with curative intent. The survival data was collected and presented according to Kaplan-Meier. Single- and multiple predictor survival analyses were carried out using the log-rank test and Cox proportional hazards model.

**Results:**

Single-predictor survival analysis showed survival to be dependent on the Masaoka-Koga classification, WHO histological classification, resection status, surgical technique, and Clavien-Dindo grade for postoperative complications. Multiple predictor analysis confirms that the Masaoka-Koga stage (HR = 4.876, *P* = 0.032) and Clavien-Dindo grade (HR = 4.904, *P* = 0.011) are independent prognostic factors for survival.

**Conclusion:**

In addition to the Masaoka stage, the occurrence of severe postoperative complications represents an independent prognostic factor. Given the tumor’s sensitivity to radiation, the use of neoadjuvant radiotherapy can be considered to downstage Masaoka-Koga stages III and higher, thus reducing surgical risks. Further prospective multicenter studies are urgently needed.

## Background

Thymic epithelial tumors are rare neoplasias. The rate of incidence is 1.7 per million per year in Europe; in the USA, it is given as 1.3 to 2.7 per million per year [1]. The disease most commonly appears between the ages of 50 and 60 [2]. The 5-year survival rate is 30 to 60% for patients with thymic carcinoma [3]. Due to its rarity, there are no internationally accepted standards or national guidelines for managing this complex oncological disease. Prospective studies are difficult owing to the infrequency of the disease. Current knowledge is based predominantly on small, retrospective single-center studies [4–6], or database analyses such as Surveillance Epidemiology and End Results (SEER) and National Cancer Data Base (NCDB). These studies often have methodological weaknesses: for instance, important confounders, such as the severity of postoperative complications, are not systematically documented. In some cases, resection status and histological classification are recorded only subsequently. Also, the included cases have heterogeneous treatment modalities, could have taken place as long as up to 50 years ago, and do not take the interim progress in surgical interventions or radiotherapy into account [7–9].

The thymus is a primary lymphoepithelial organ. It plays a central role in the maturation of thymocytes into thymus lymphocytes (T cells). Histomorphologically, the thymus can be divided into the cortex and medulla. In the cortex and medulla, finely branched epithelial cells connect with each other to form a three-dimensional network that surrounds the thymocytes. Over the course of maturation, the thymocytes migrate from the cortex to the medulla. The numerous lobes of the thymus are contained by a delicate, connective tissue-like capsule. After puberty, the specialized thymus tissue is replaced by fatty tissue, although the residual thymus tissue continues to remain indefinitely [10].

Thymomas originate from the epithelial cells of the thymus. Thirty to 40% of patients with thymoma develop symptoms of myasthenia gravis. Clinical signs entail drooping eyelids, double vision, and rapid exhaustion. Conversely, only five to 15% of all patients with myasthenia gravis have thymomas. In contrast to thymoma, there are practically never clinical signs of myasthenia gravis in the case of thymic carcinoma [11].

Transcapsular invasion is used as a criterion to differentiate histologically between thymoma and thymic carcinoma. In 1981, Masaoka et al. stated that the clinical course depends on the invasion of the capsule and the surrounding structures as well as pleural, pericardial, lymphogenous, and hematogenous metastases [12]. This led to the definition of four stages, which were then specified even further by Koga et al. in 1994 [13]. This staging system is currently recommended by the International Thymic Malignancy Interest Group to classify patients. It is the one most often used worldwide (Table 1) [14, 15]. No less established in clinical practice is the World Health Organization (WHO) histological classification. Marx et al. validated six subtypes based on tumor morphology and the ratio of lymphocytes to epithelial cells (Table 2) [16].

**Table 1:** Classification of thymomas according to Masaoka-Koga stage [14]

Stage	Masaoka-Koga staging system
I	Grossly and microscopically completely encapsulated tumor
II a	Microscopic transcapsular invasion
II b	Macroscopic invasion into thymic or surrounding fatty tissue, or grossly adherent to but not breaking through mediastinal pleura or pericardium
III	Macroscopic invasion into a neighboring organ (i.e., pericardium, great vessel, or lung)
IV a	Pleural or pericardial metastases
IV b	Lymphogenous or hematogenous metastasis

**Table 2:** Types of thymic tumors according to WHO histological classification [16]

WHO type	Histologic description
A	Medullary thymoma
AB	Mixed thymoma
B1	Predominantly cortical thymoma
B2	Cortical thymoma
B3	Well-differentiated thymic carcinoma
C	Thymic carcinoma

The disease is often detected late if no typical signs of myasthenia are present. Among the symptoms are localized feeling of pressure, retrosternal pain, and coughing. Large tumors can lead to superior vena cava syndrome. The phrenic nerve or the recurrent laryngeal nerve can be infiltrated and result in neurological deficiencies. The diagnostic standard is CT with contrast agent. Performing an MRI or PET-CT can make sense to determine resectability and exclude distant metastasis [17, 18]. No preoperative biopsy is required if the tumor is deemed to be fully resectable based on such imaging.

The recommended therapy for patients with a thymic epithelial tumor currently depends on whether the tumor appears to be resectable prior to surgery. If this is the case, complete excision is the primary therapy. Anatomically, the thymus is located in the anterior mediastinum. The ventral border is the sternum. Dorsally, the thymus is positioned on the pericardium, the aorta, the superior vena cava, and the brachiocephalic veins. An extended resection is required if the tumor has spread into these critical mediastinal structures or into the lung [19]. If complete resection is not achievable, neoadjuvant therapy can be attempted to downstage or alternatively debulking surgery with subsequent adjuvant treatment [6, 20]. In terms of surgical techniques, minimally invasive procedures and conventional open surgery are available [21–23]. Minimally invasive techniques are limited by infiltrative tumor growth into adjacent organs; complete excision is always the outcome aimed for.

Thymic epithelial tumors are radio-sensitive [24], so if resection is incomplete, there is a clear indication for adjuvant radiotherapy. In the case of an R0 resection, the benefit of postoperative radiotherapy depends on tumor stage [7, 25]. There is consensus that adjuvant radiotherapy is worthwhile in patients with Masaoka-Koga stages III and higher, regardless of resection status. Palliative chemotherapy is indicated in the event of metastatic cancer. An optimal chemotherapy regimen has not yet been established [26, 27]. Usually, a combination of cisplatin, doxorubicin, and cyclophosphamide is used [1, 28]. The molecular basis of this cancer is only rudimentarily understood. Targeted therapies are the subject of current studies [1].

Given this problematic context, it is absolutely necessary to report current experiences and survival data regarding these rare tumor entities and, in doing so, to contribute to validating and developing our current therapeutic approach.

## Methods

### Study population

The retrospective study included 40 patients with primarily resectable thymic epithelial tumor who underwent resection with curative intent between 2004 and 2018 at the Department of General, Visceral and Thoracic Surgery at the University Medical Centre Hamburg-Eppendorf. None of the patients received neoadjuvant therapy. The tumor stage was identified according to Masaoka-Koga [13]. Tumor histopathology was classified in compliance with WHO [16]. Postoperative complications were classified according to Clavien-Dindo (Table 3) [29]. The overall survival (OS) was computed as the time period from the date of surgery to either the date of death or last follow-up, whichever occurred first. Events considered for survival analysis were death due to cancer diagnosis. If no event was recorded, the patients were censored at the last contact for statistical evaluation. The median follow-up period for the patient group was 70.5 months. The study protocol was approved by the Ethics Committee of the Medical Board in Hamburg, Germany. Informed written consent was obtained from all participants.

**Table 3:** Classification of complications according to Clavien-Dindo

Grade	Definition
I	Any deviation from the normal postoperative course without the need for pharmacological treatment or surgical, endoscopic, and radiological interventions. Allowed therapeutic regimens are drugs such as antiemetics, antipyretics, analgetics, diuretics, and electrolytes and physiotherapy. This grade also includes wound infections opened at the bedside.
II	Requiring pharmacological treatment with drugs other than such allowed for grade I complications. Blood transfusions and total parenteral nutrition are also included.
IIIa	Requiring surgical, endoscopic, or radiological intervention: Intervention not under general anesthesia
IIIb	Requiring surgical, endoscopic, or radiological intervention: Intervention under general anesthesia.
IV a	Life-threatening complication (including CNS complications) requiring IC/ICU-management: single-organ dysfunction (including dialysis)
IV b	Life-threatening complication (including CNS complications) requiring IC/ICU-management: multiorgan dysfunction
V	Death of a patient

### Statistical analysis

The *Statistical Package for Social Sciences (SPSS®*) for Mac (Version 25) (IBM, Armonk, NY, USA) was used for the statistical analysis. Descriptive statistics were used to describe patient baseline characteristics. The chi-square test was used for correlation between discrete variables. Survival curves for the overall survival of the patients were plotted (Kaplan-Meier method) and analyzed by implementing the log-rank test as a single-predictor model. Cox proportional hazard regression was performed as a multiple predictor model of survival. The interactions between the time measure and the covariates were tested in the multiple predictor model. None of the covariates was time-dependent (*P* > 0.05). Therefore, the proportional hazard assumption was confirmed. Statements of significance refer to *P* values of two-tailed tests that were less than 0.05.

## Results

The study population consisted of 26 (65%) women and 14 (35%) men. The median age was 55.2 years (range 30–85). The 5-year survival for all included patients was 70.5%. The included patients had the following Masaoka-Koga stages: stage I, 10 (27%); stage IIa, 14 (37.8%); stage IIb, 1 (2.7%); stage IVa, 5 (12.5%); and IVb, 3 (7.5%). Pleural and/or pericardial metastases of the tumor were seen in stage IVa patients. In patients with stage IVb, there was tumor infiltration of the lymph nodes, lungs, and/or heart. All included patients underwent resection with curative intent. An R0 resection was achieved in 27 (67.5%) patients. Half of the included patients underwent minimally invasive surgery, while open surgery was selected for the remaining patients. In 11 (27.5%) patients, there was an R1 situation, and in two (5 %) patients an R2 situation. The postoperative complications were classified according to Clavien-Dindo: grade I, 18 (45%); grade II, 10 (25%): grade IIIa, 4 (10%); grade IIIb, 5 (12.5%); grade IVa, 2 (5%); and grade V, 1 (2.5%). One patient died during the postoperative hospital stay. The postoperative complications are listed in detail in Table 4. Adjuvant radiotherapy was administered to 15 (37.5%) patients. The total dose varied between 50 and 60 Gy. Eight (20%) of the included patients received platinum-based adjuvant chemotherapy. Four (10%) patients received adjuvant chemotherapy and radiotherapy.

**Table 4:** Frequency of postoperative complications

Complication	No.	%
Respiratory failure requiring reintubation	2	5
Pneumonia	4	10
Pneumothorax	2	5
Thrombosis of the superior vena cava	2	5
Postoperative bleeding	4	10
Pericardial tamponade	1	3
*C. difficile* infection	2	4
Catheter-related bloodstream infection	1	3
Systemic inflammatory response syndrome	2	4
Myasthenic crisis	2	4
Recurrent laryngeal nerve paralysis	1	3
Exitus letalis	1	3

### Single-predictor survival analysis

**Fig. 1:**
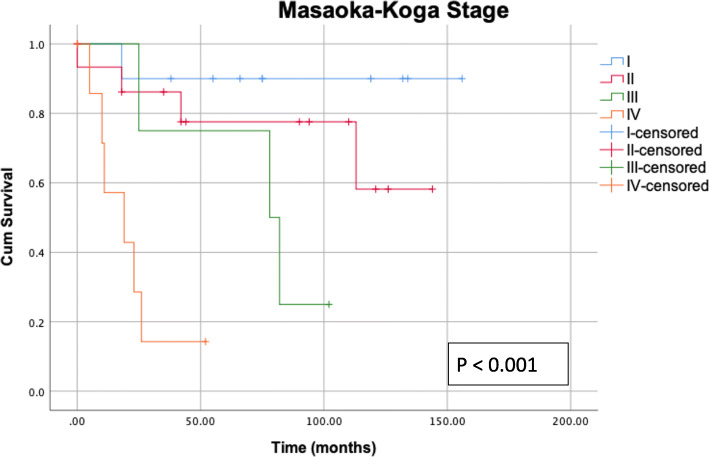
Kaplan-Meier survival curves plotted according to Masaoka-Koga. Classification according to Masaoka-Koga enables a highly significant stratification of survival (*P* < 0.001, log-rank test)

The results of the single-predictor survival analysis are summarized in Table 5. Gender and age exert no influence on survival. Classification according to Masaoka-Koga enables a sufficient stratification of survival (*P* < 0.001, Fig. 1). Patients with WHO histologic description B3 (well-differentiated thymic carcinoma) or C tumor (thymic carcinoma) show poorer survival than patients in the other groups: A (medullary thymoma), AB (mixed thymoma), B1 (predominantly cortical thymoma), and B2 (cortical thymoma) (*P* = 0.007). Patients who underwent minimally invasive surgery have better survival than patients who underwent conventional open surgery (*P* = 0.001). Patients with an advanced Masaoka-Koga stage more often underwent open surgery (*P* = 0.033, Table 6).

**Table 5:** Patient characteristics and a single-predictor analysis of overall survival

Characteristics	No.	%	Mean survival (95 % CI)	*P* (log-rank test)
Gender				0.870
Female	26	65	119 (95–144)	
Male	14	35	118 (76–161)	
Age, years				0.755
≤ 60	26	65	121 (93–151)	
> 60	14	35	105 (70–141)	
Masaoka-Koga stage				**< 0.001**
I	10	27	142 (117–168)	
II	15	41	111 (83–138)	
III	4	11	72 (44–100)	
IV	8	22	21 (10–32)	
Surgical technique				**0.001**
Minimally invasive	20	50	162 (139–185)	
Open	20	50	79 (48–111)	
Resection status				**0.012**
R0	27	68	142 (115–168)	
R1	11	28	71 (40–102)	
R2	2	5	23 (16–29))	
Adjuvant radiotherapy				0.578
Yes	15	37	84 (60–109)	
No	25	62	126 (96–156)	
Adjuvant chemotherapy				**0.001**
Yes	8	20	38 (14–62)	
No	32	80	138 (113–162)	
Clavien-Dindo classification				**0.012**
I–IIIa	32	80	135 (109–161)	
IIIb–IV	8	20	55 (20–90)

**Table 6:** Baseline characteristics: treatment and complications (chi-square test)

	Surgery technique			Adjuvant chemotherapy			Adjuvant radiotherapy			Clavien-Dindo		
Characteristics	Minimally invasive	open	*P*	Yes	No	*P*	Yes	No	*P*	I–IIIa	IIIb–IV	*P*
Gender			1			0.868			0.608			0.868
Female	13	13		5	21		9	17		21	5	
Male	7	7		3	11		6	8		11	3	
Age, years			0.440			0.507			0.123			0.868
≤ 60	14	12		6	20		12	14		21	5	
> 60	6	8		2	12		3	11		11	3	
Masaoka stage			**0.033**			**< 0.001**			**0.019**			0.256
I	7	3		0	10		1	9		10	0	
II	10	5		1	14		7	8		10	5	
III	1	3		1	3		0	4		3	1	
IV	1	7		6	2		5	3		6	2	
Surgery technique			–			0.114			0.744			**0.018**
Minimally invasive	–	–		2	18		7	13		19	1	
Open	–	–		6	14		8	12		13	7	
Residual tumor			0.938			**< 0.001**			0.331			0.637
R0	14	13		1	26		8	19		22	5	
R1	5	6		5	6		6	5		8	3	
R2	1	1		2	0		1	1		2	0	
Adjuvant radiotherapy			0.744			0.410			–			0.414
Yes	7	8		4	11		–	–		11	4	
No	13	12		4	21		–	–		21	4	
Adjuvant chemotherapy			0.114			–			0.414			0.553
Yes	2	6		–	–		4	4		7	1	
No	18	14		–	–		11	21		25	7	
Clavien-Dindo			**0.018**			0.553			0.414			
I–IIIa	19	13		7	25		11	21		–	–	
IIIb–IV	1	7		1	7		4	4		–	–	

**Fig. 2:**
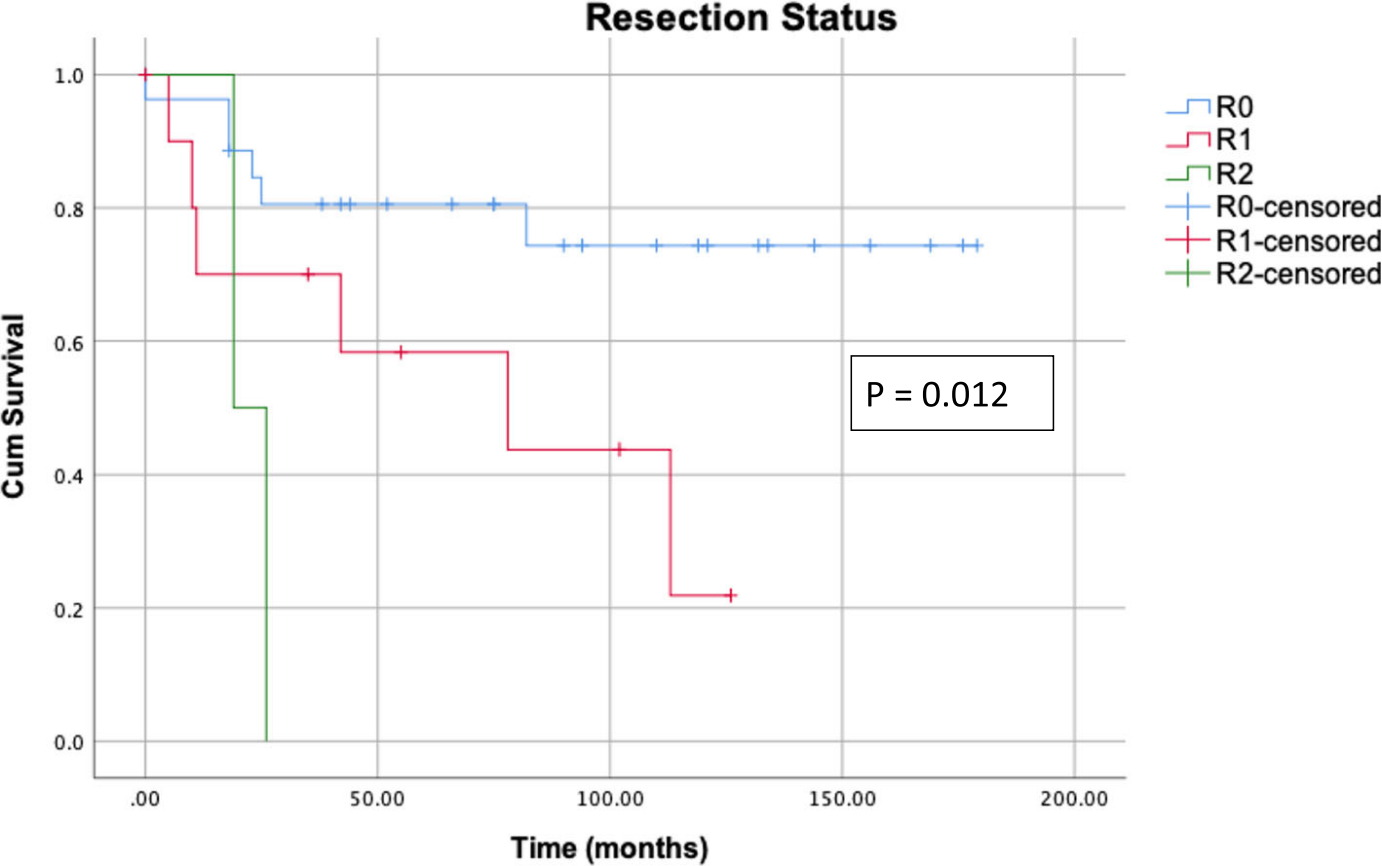
Kaplan-Meier survival curves plotted according to resection status. Patients with incomplete tumor resection show poorer survival (*P* = 0.002)

**Fig. 3:**
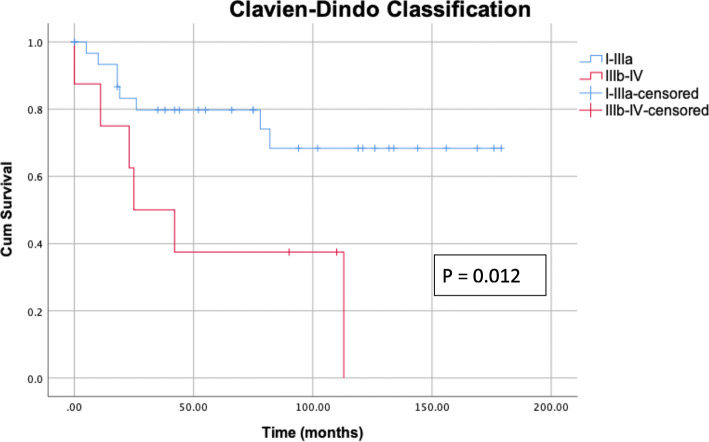
Kaplan-Meier survival curves plotted according to the severity of postoperative complications (Clavien-Dindo grade). Severe complications, in particular those classified as grade IIIb, are accompanied long term by a distinctly reduced survival (*P* < 0.001)

Resection status correlates with survival. Patients with incomplete tumor resection show poorer survival (*P* = 0.012, Fig. 2). Comparing the survival of patients with R0 and R1 resection only, excluding patients with R2 resection, a better survival for patients with R0 resection can be confirmed (*P* = 0.032). There is a highly significant correlation between the postoperative complications classified according to Clavien-Dindo and survival. Severe complications, particularly grade IIIb (intervention under general anesthesia), are accompanied long term by a distinctly reduced survival (Fig. 3). Severe complications occur more often if open surgery had been performed (*P* = 0.018, Table 6).

Patients who received postoperative radiotherapy show no significant difference in survival in comparison to patients who received none (*P* = 0.578). More advanced Masaoka-Koga stages were more frequent in the group of patients who received postoperative radiotherapy (*P* = 0.019, Table 6). Chemotherapy was more often carried out if there was an advanced Masaoka-Koga stage (*P* < 0.001, Table 6). Patients who received adjuvant chemotherapy show significantly poorer survival (*P* = 0.001, Table 5). Incomplete resection was present in 88% of the patients who received adjuvant chemotherapy (*P* < 0.001, Table 6).

### Multiple predictor survival analysis

Multiple predictor survival analysis looks at the parameters of the Masaoka-Koga stage (I + II vs. III + IV), complete resection (yes vs. no), Clavien-Dindo grade (I–IIIa vs. IIIb–IV) and adjuvant chemotherapy (no vs. yes). With an HR of 4.876 [95% Cl 1.145; 20.775], the Masaoka-Koga stage is an independent prognostic factor (*P* = 0.032). Hence, the risk of death for patients with the advanced Masaoka-Koga stage increases 4.876-fold in comparison to patients with early diseases (Table 7). Likewise, the degree of severity of the postoperative complication stratified according to Clavien-Dindo shows itself to be an independent prognostic factor with an HR of 4.904 [95% Cl 1.447; 16.623] (*P* = 0.011). Thus, the risk of death for patients with severe complications increases 4.904-fold. No other included parameter attains significance (Table 7).

**Table 7:** A multiple predictor survival analysis for overall survival

	HR	95 % Cl for HR	*P*
Masaoka-Koga stage (I + II vs. III + IV)	**4.876**	1.145–20.775	**0.032**
Complete resection (yes vs. no)	1.477	0.419–5.204	0.544
Clavien-Dindo (I–IIIa vs. IIIb–IV)	**4.904**	1.447–16.623	**0.011**
Adjuvant chemotherapy (no vs. yes)	2.803	0.530–14.816	0.225

## Discussion

In the present study, we analyzed the treatment and survival of patients with thymic epithelial tumors taking relevant prognostic factors into account, including the severity of postoperative complications.

Classification according to the Masaoka-Koga staging system shows itself to be valid: the more advanced the Masaoka-Koga stage, the poorer the survival. The multiple predictor analysis confirmed the Masaoka-Koga stage as an independent prognostic factor (HR = 4.876 [95% Cl 1.145; 20.775], *P* = 0.032). This corroborates the literature in which the Masaoka-Koga stage is established as a suitable parameter for estimating the prognosis [3, 30, 31]. Likewise, the diagnostically complex WHO histologic description can be confirmed by the single-predictor analysis as a prognostic factor for survival. Patients with incomplete tumor resection show a poorer survival, which is consistent with many studies [32]. Gender has no influence on survival. Weksler et al. report that gender is a prognostic factor; however, this cannot be confirmed based on the data presented here [3].

The multiple predictor analysis of our study shows that a complication corresponding to Clavien-Dindo grade IIIb or higher is an independent prognostic factor for survival (HR = 4.904 [95% Cl 1.447; 16.623], *P* = 0.011). Severe complications occurred significantly more often if an open surgery was performed. This type of operation was more frequently necessary in the advanced Masaoka-Koga stages [19]. In the context of other surgical procedures, e.g., colectomy, lobectomy, and pneumonectomy, the impact of postoperative complications on survival has already been demonstrated [33]. However, studies on which current treatment of thymic epithelial tumors is based have not investigated the influence of postoperative complications on long-term survival [4–9]. Consequently, the severity of postoperative complications must be considered in future prospective studies.

Thymic epithelial tumors are radio-sensitive [24]. In the event of incomplete tumor excision, there is a clear indication for adjuvant radiotherapy. Consensus exists on the performance of adjuvant radiotherapy for the Masaoka-Koga stages III and higher, regardless of resection status. Patients in this study who received adjuvant radiotherapy showed no difference in survival compared to those who received none. It must be noted here that patients who did receive radiation had a significantly more advanced Masaoka-Koga stage (*P* = 0.019, Table 6). Thus, it can be assumed that adjuvant radiotherapy improved the prognosis of patients with the advanced Masaoka-Koga stages. Ahmad et al. demonstrated that the benefit of postoperative radiotherapy depends on tumor stage [7, 25]. Due to the rarity of the investigated tumor entity, the number of patients included in this study is too small for subgroup analysis according to the Masaoka-Koga stage. Besides, the small number of patients results in relatively wide confidence intervals of the hazard ratios. The resulting statistical uncertainty is, along with the retrospective nature, a limitation of this study.

## Conclusion

The postoperative course in patients with an advanced Masaoka-Koga stage (stages III–IV) is more often characterized by severe complications resulting from the necessarily more extensive surgery. In the multiple predictor analysis, severe postoperative complications (Clavien-Dindo grade ≥ IIIb) represent an independent prognostic factor for survival. Future studies must, therefore, systematically document complications that occur following surgery. The efficacy of postoperative radiotherapy can be confirmed by these study findings. Neoadjuvant radiotherapy for Masaoka-Koga stage III and higher could initially lead to downstaging and increase the probability of complete resection using minimally invasive procedures, thereby reducing the extent of resection. Consequently, the frequency of severe postoperative complications and the accompanying disadvantage to survival could be reduced. Initial studies by Lucchi et al. and Korst et al., who have already tested this hypothesis, demonstrate the feasibility of this therapeutic strategy [20, 34]. Twenty-two patients were included in the prospective multicentric study by Korst et al. In that study, 11 of the 22 patients were in regression after undergoing neoadjuvant therapy with a consecutive high percentage of complete resections. Our data support that additional prospective multicenter studies are urgently needed to evaluate this therapeutic approach, which, due to the rarity of this disease, can only be realized as multicentric studies.

## Data Availability

The datasets used and analyzed during the current study are available from the corresponding author on reasonable request.

## Abbreviations

SEER: Surveillance epidemiology and end results
NCDB: National cancer data base
T cells: Thymus lymphocytes
WHO: World health organization
CT: Computed tomography
MRI: Magnetic resonance imaging
PET-CT: Positron emission tomography-computed tomography
SPSS®: Statistical package for social sciences
